# Instrumental Diagnosis—Sphymography

**Published:** 1868-07

**Authors:** Philip S. Wales

**Affiliations:** Surgeon U. S. Navy


### Instrumental Diagnosis—Sphymography.

BY PHILIP S. WALES, M. D., SURGEON U. S. NAVY.

The sphygmograph, derived from two Greek words, signifying *pulse* and *pen*, was first applied in physiological researches upon the circulation of the blood, but recently has been turned into another channel—the investigation of disease.

The difficulty of accurately appreciating and estimating the properties or qualities of the pulse in health and disease by the unassisted senses, has always stood in the way of progress in the knowledge of the value of its indications, and therefore has prevented the attainment of much accurate and reliable prognostic and diagnostic information from its examination in disease. The tactile power, by means of which we gain such knowledge, is, however, so various in different persons, that with the same pulse different impressions as to its qualities of fulness, force and volume, may be conveyed to them, and therefore different conclusions will be arrived at, though the conditions of the observation be the same. To appreciate the innumerable variations of the pulse, and their import in prognosis and diagnosis, a long individual training is absolutely required, when the sense of touch is alone employed in the investigation. Even after this knowledge has been acquired, it is of that sort of individual possession which cannot be transmitted to another, or taught by any form of words you choose to adopt, while the tactile power is so various, except in the most imperfect manner.

From this imperfection of the sense of touch in accurately determining the character and import of the pulse qualities, the working physician will gladly welcome any extrinsic aid from physics, in giving more certainty to his observations and deductions in the daily pursuit of his profession. This important desideratum is now promised him in the sphymograph.

As early as 1837, King, of London, in order to make more evident the motion of certain veins presenting the phenomenon of rhythm, employed a lever for amplifying its extent. Later, Vierordt, a German physiologist, applied the same happy idea to the ascertainment of arterial rhythm, and by means of the lever was enabled to make evident to the eye in the form of diagramatic sketches, the qualities of arterial action; but the great objection to his instrument is that the lever is too heavy, and so poised that its tr cings were too uniform, and the component lines nearly vertical and parallel under all variations in the arterial current, and so far they did not represent the various pulse-forms correctly, or possess sufficiently distinctive and characteristic properties as to enable the physician to draw any conclusions as to the various morbid states of the circulating and other organs.

With a view of correcting this manifest imperfection in the instrument as a diagnostic mean, Dr. E. J. Marey, of Paris, well known for his scientific attainments as a physiologist, and as an acute observer, modified it in many material points, so as to produce almost an altogether new instrument. He simply retained the lever motion of the instrument of Vierordt to multiply the extent of arterial action, and then appended other portions to it, so that the complete apparatus worked in such complete harmony, and with such accuracy, as to enable him to present to the eye the most faithful representations of the pulse-forms under the most varying conditions of disease.

A metallic plate, furnished with movable lateral wings, forms the frame of the instrument, and is in the shape of a section of a hollow cylinder, the concavity of which may be altered at pleasure to fit it accurately to the fore-arm. At the sides of this frame there are six hooks, three upon a side, which enable the instrument to be fastened to the part securely by means of a cord passing around them alternately from side to side. The end of a steel spring is attached to the back part of the plate, and projects forward to the position of the artery, being brought perpendicularly in contact with it by a little ivory plate interposed between them. The motion of the artery is transferred from this steel spring to the tracing lever, by a metallic stem bent upon itself placed between them, connected by its long arm to the spring and touching the lever near its centre of motion, which is at the anterior edge of the metallic frame, by the distal end of its short arm; the relation of these three parts to each other and to the artery are regulated by two milled-headed screws. The tracing lever carries at its distal end a pen which marks upon a paper, supported upon the oblong plate the tracings; the paper is moved its whole length every ten seconds, as the writing goes on, by the watch work, which is wound up by a button key, and started or stopped at will by a regulator placed at the side of the instrument.

Variation of arterial tension, which is the agency in producing the difference in the pulse-form, is due, according to Dr. Marey, in a great measure, to causes which may be arranged in two principal groups. In the first, are those causes which facilitate the passage of the blood through the capillaries, thereby inducing a *low arterial tension;* in the second, those which obstruct its course in the capillaries giving rise to a *high tension.* The physical condition culminating in these variations of tension are, according to these views, 1st, either mechanical, as the alteration of the position of the subject, and by compressing the arteries so as to arrest the free flow of the blood through them; 2d, or by causing the construction or relaxation of the smaller vessels so as to obstruct the blood in its course to the veins. Every cause which contracts them, as for instance, the application of cold will result in an *elevation* of the arterial tension; every cause which relaxes them, as warmth will *lower* the tension. It follows from all this, that the principal conditions for alterations of tension are induced by causes acting upon the periphery of the animal body.

The pulse-forms corresponding with these two states of arterial tension, are well marked and characteristic. With a low tension the first element of the pulsation, or the *line of ascent*, will be steep and ample, while the third element or *line of descent*, will present the form of undulations.

With a strong tension the characters of the tracings will present a strong contrast with the preceding; the line of ascent will be less long and abrupt, while the line of descent will move off obliquely in a straight line.

The above two traces show that the frequency of the heart's action is also affected according as the tension is greater or less; being more frequent with less tension and the reverse.

Two forces, *systolic acceleration* and *increased arterial tension*, are transmitted through the arterial system, and acting simultaneously and conjointly, produce the pulsations in the aorta and great vessels, at the root of the neck. Passing further on in the divisions and sub-divisions of the vessels, these forces are no longer coincident, and separate more and more as they proceed.

Pulse-traces taken at a time when ventricular contraction and natural tension are active and physiological, present a number of continuous curves, each representing a complete cardiac revolution. Each curve consists of three parts—a *line* of *ascent*, a *summit*, and a *line* of *descent*, which require individual notice, inasmuch as they are the factors which varying in character as to form, length, regularity and height, furnish the indication, in their different combinations, of the state of the circulation in health and disease.

The line of ascent is produced by the systolic contractions of the ventricle, which propagates an instantaneous pressure-wave from the heart to the artery upon which the sphygmograph is placed. This line varies in height, according to the facility with which the pressure-wave overcomes the arterial tension—being greater, if this is easily effected, and less under reverse circumstances. The position of the line as to verticality is determined by the rapidity of the efflux of blood into the arteries; the greater this is, the more quickly will arterial tension be established and nearer will the line approach a vertical position. When the entry of the blood is languid the line is described in an oblique direction and sometimes in a curved one.

The first change in the direction of the tracing lever forms the summit of the curve. It corresponds to that period when the blood is passing into the artery and thence onward, or when the *afflux* and *efflux* mutually balance each other. According as this period is longer or shorter, the summit will present either a point or a line of some height; in the latter case if the afflux predominates, instead of a horizontal line, we should have one with an upward slope, and under reverse circumstances one with a downward slope.

The line of descent presents a greatly waved form with three deviations form a reetilinear course, reckoning the apex of the curve as the first. The second wave represents an expansible movement in the arterial walls, and marks the termination of the systolic portion of one cardiac revolution. The notch following the second wave answers to the period of closure of the semilunar valves. The third wave succeeding the aortic notch is caused by a renewal of the action of the arterial walls. After describing the third wave, the tracing bar falls until it reaches the point which marks the period of *minimum* arterial pressure. In the normal condition of the circulation a straight line should just touch the pieces of the successive curves, which indicate the points of *maximum* tension, and runs parallel with a similar line connecting their bases, which are the points of *minimum* tension.

In certain cardiac diseases and in febrile derangements, the second wave is effaced, the aortic notch deepens, and the third wave is somewhat exaggerated, thus a two-waved line of descent is formed and the pulse-form is said to be *dicrotic*. Dicrotism varies in degree with the nature and intensity of the febrile disease; if the bottom of the aortic notch reaches half way to the base-line of the curve, the pulse is designated *subdicrotous;* if the notch touches the base line, *full dicrotism* is established; and in exceedingly bad cases of fever the notch extends below this line giving a *hyperdicrotic* pulse-form.

The pulse-forms of valvular disease will vary very much, according to the nature of the alteration and its situation. These morbid conditions are rarely single, but are associated in various degrees of complexity, and the pulse-forms must necessarily be also complexed in their forms; these may, however, be analyzed into their simple forms, and these forms connected with special anatomical changes.—*N. O. Journal of Medicine.*

